# The Association between Vitamin D and the Components of Male Fertility: A Systematic Review

**DOI:** 10.3390/biomedicines11010090

**Published:** 2022-12-29

**Authors:** Daria Adamczewska, Jolanta Słowikowska-Hilczer, Renata Walczak-Jędrzejowska

**Affiliations:** Department of Andrology and Reproductive Endocrinology, Medical University of Lodz, 92-213 Lodz, Poland

**Keywords:** vitamin D, supplementation, male fertility, semen quality, sex hormones

## Abstract

Objective: Previous systematic reviews of the effects of vitamin D on the components of male fertility have been inconclusive. This article systematically reviews the latest research to examine the relationship between vitamin D, semen quality parameters, and sex hormones production. Methods: MEDLINE, Cochrane, and Web of Science databases were searched using the appropriate keywords. Results: Observational studies indicate significant correlation between vitamin D levels and sperm parameters, with a particular emphasis on sperm motility, and partially suggest a relationship between higher serum testosterone and vitamin D levels. Additionally, interventional studies confirmed that vitamin D supplementation has a positive effect on sperm motility, especially progressive. However, most randomized clinical trials indicate that vitamin D treatment does not have any significant effect on testosterone or other hormone levels. Conclusions: Although our findings add to the discussion regarding the effect of vitamin D on male fertility, there is still no solid evidence to support the use of vitamin D supplementation to improve the outcomes of patients with impaired sperm parameters and hormonal disorders. Additional dedicated clinical studies are needed to clarify the relationship between vitamin D and male fertility, along with its components.

## 1. Introduction

The male gonads perform two very important functions that are essential for the male reproductive system; they produce gametes, i.e., spermatozoa, and release sex hormones [1]. Their proper functioning depends on many different factors: age, the influence of nutrition, stimulants, hormones, pharmacological agents, and substances that disrupt the hormonal and vitamin balance [2]. Currently, the research on male reproductive health is focusing on the growing global trend of declining sperm count and increasing male reproductive system abnormalities, which has been related, among others, to lifestyle factors as well as environmental impact. Among these, numerous studies have investigated the role of vitamin D (VD) in the proper functioning of the male reproductive system.

Initially, the action of VD was associated mainly with the maintenance of calcium and phosphorus homeostasis, which have a significant effect on skeleton mineralization. However, in recent years, new knowledge has emerged about its biological function and its potential to reduce the risk of many chronic diseases [3]. The known range of its target organs has expanded, and the discovery of vitamin D receptor (VDR) expression and vitamin D metabolizing enzymes (VDME) in the testes, male reproductive tract, and human sperm suggests that VD plays a role in the male reproductive role [4]. Although several reviews have been made on the subject, no consensus has been reached regarding the role of VD in male fertility. Moreover, new intervention studies in humans have only appeared in the last few years

The aim of this paper is to provide an up-to-date, comprehensive review of the existing literature regarding the experimental and clinical evidence for the effects of VD on the components of male fertility, sperm parameters, and sex hormone production.

## 2. Background

### 2.1. Vitamin D Physiology

**Figure 1:**
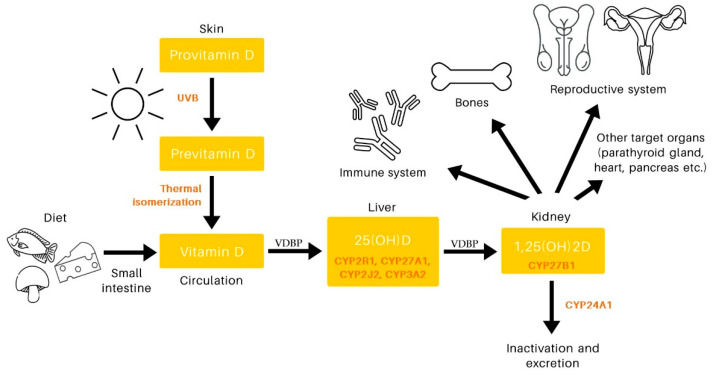
Overview of the vitamin D pathway. The main endogenous source of vitamin D is sunlight, which stimulates the synthesis of previtamin D3. Vitamin D can also be absorbed in the intestines from food. From the skin or intestines, vitamin D goes into the blood where it is bound to protein. It is then hydroxylated in the liver and kidneys to produce 1,25-dihydroxyvitamin D, a physiologically active form of vitamin D that functions in many parts of the body, including the skeletal system, immune system, reproductive system, and others. Abbreviations: CYP: cytochrome P-450; UVB: ultraviolet B; VDBP: vitamin D-binding protein.

VD is a fat-soluble vitamin based on a sterol ring. There are two forms of VD: vitamin D2 (ergocalciferol, VD2), found in plants and fungi, and D3 (cholecalciferol, VD3), produced in animals. In humans, it can be obtained through the diet, but about 90% of the VD detectable in human serum is synthesized in the skin from a precursor (provitamin D3) present in epidermal cells. The provitamin D3 is transformed into provitamin D3 under the influence of solar ultraviolet B radiation (wavelength 290–315 nm), and then it is converted to VD3 by heat-dependent isomerization [5,6] (Figure 1). The VD produced in the skin enters the circulation and is transported to the liver by a protein called vitamin D-binding protein (VDBP). In contrast, VD from the diet is absorbed in the small intestine, where it binds to chylomicrons, which are then released into the lymphatic system and enter the venous circulation. These chylomicrons then bind with VDBP and reach the liver [7].

Although VD is classified as a vitamin, in practice it is a prohormone, because in the body it is transformed into a biologically active form, 1alpha,25-dihydroxyvitamin D, 1,25(OH)2D. The first stage of conversion to its active form takes place in the liver. Vitamin 25(OH)D—calcidiol—is produced by enzymatic hydroxylation of a 25-carbon molecule by one of the four hepatic cytochrome P-450 enzymes. Three of them are microsomal forms, CYP2R1, CYP2J2, and CYP3A4. The fourth enzyme CYP27A1 is mitochondrial [8,9]. 25(OH)D is transferred, in combination with VDBP, from the liver to the kidneys, where CYP27B1 creates another form of VD, calcitriol, 1,25(OH)2D [3,10]. Calcitriol is the hormonally active form of VD that is responsible for most of its biological effects.

Calcitriol acts on various cells of the body, mainly through the VDR located in the cell nucleus. The VDR is a nuclear receptor that belongs to the superfamily of steroid hormone receptors, which includes receptors for retinoic acid, thyroid hormone, sex hormones, and adrenal steroids. The VDR is made up of three main domains: a linker, a DNA binding unit, and a ligand binding unit.

Calcitriol can act through VDR by genomic (classical) and non-genomic pathways [11]. In the former, 1,25(OH)2D attaches to a nuclear VDR, which then binds to the retinoid X receptor (RXR). The resulting VDR-RXR heterodimer functions as a transcription factor—it binds to a specific DNA sequence present in the promoter regions, referred to as the vitamin D response element (VDRE), which induces or inhibits the expression of target genes [12]. Advanced genomic techniques such as ChIP-seq have shown that VDR has thousands of genome-wide binding sites influencing the transcription of hundreds of different genes. In a study with human lymphoblastoid cell lines treated with 1,25(OH)2D, 2776 VDR binding sites were found to alter the expression of 229 genes [13]. In contrast, the latter, non-genomic pathway involves the activation of signaling molecules such as phospholipase C and phospholipase A_2_, phosphatidylinositol-3 kinase and p21ras, and the rapid generation of second messengers (Ca^2+^, cyclic AMP, fatty acids), accompanied by the activation of protein kinases, such as protein kinase A, src, mitogen-activated protein kinases, protein kinase C (PKC), and Ca^2+^-calmodulin kinase II [14,15,16,17,18,19]. It also includes the opening of Ca^2+^ and Cl^−^ channels [20]. It has also been recognized that the VDR can mediate rapid non-genomic actions when located in the membrane or cytoplasm [21].

The circulating forms of VD are inactivated in the kidneys and in several target organs [22]. Ultimately, through a series of successive reactions catalyzed by the enzyme 24-hydroxylase (identified as CYP24A1), 1,25(OH)2D is converted into water-soluble calcitric acid, which can be excreted in the urine [23,24].

The level of VD storage in the human body is best reflected by the concentration of 25(OH)D in the serum; 1,25(OH)2D assessment is of limited utility [25,26]. The North American Institute of Medicine (IOM) Dietary Reference Committee (DRI), in cooperation with UK and European Union authorities, established dietary reference values for VD in adults using serum 25(OH)D levels as an indicator of VD status [27]. They came to the conclusion that a serum level of 20 ng/mL is essential for bone health [28]. However, other medical expert committees disagreed, claiming that a value of at least 30 ng/mL is required to prevent skeletal and other system diseases [29,30,31].

### 2.2. Vitamin D in Male Reproductive System

In the early days of research into the metabolism of VD, the kidney was thought to be the only site of 1,25(OH)2D production. While the kidney is the primary endocrine organ that produces 1,25(OH)2D in response to other calcium- and phosphate-regulating hormones, it is now known that other organs also have the ability to convert 25(OH)D to 1,25(OH)2D [24]. Subsequently, the discovery of the action of VDME and the expression of VDR in the male reproductive system suggested that VD plays a role in male fertility. One of the first significant discoveries was the detection of VDR in human testicular tissue homogenates using titrated VD [32]. In 2006, the presence of VDR was confirmed in human sperm, with VDR expression being observed predominantly in the head and nucleus of fertile men’s sperm, with some localization in the neck region [33]. An investigation, based on electron microscopy and gold labeling, found most of the distribution to occur in the nucleus, with just a minor quantity of staining in the neck [34]. The presence of nuclear VDR in terminally differentiated, transcriptionally inactive cells, such as sperm, suggests that VDR may be a remnant of late spermatogenic processes and may be associated with non-testicular sperm maturation. On the other hand, the presence of VDR can act as a mechanism of genome stability and protection against DNA strand breaks [34,35]. These hypotheses require further research.

Studies on the expression of the VDR and VDME indicate the presence of VDR, CYP2R1, CYP27A1, CYP27B1, and CYP24A1 in human testes, epididymis, prostate, seminal vesicles, and mature sperm. The activity of VDME regulates VD intracellular concentration and, thus, the activation of the VDR. For this reason, local expression of VDME is crucial. Germ cells of adult testes such as spermatogonia, spermatocytes, and round and elongated spermatids primarily express VDRs and metabolizing enzymes, indicating that local regulation of active VD may be important for spermatogenesis and/or sperm function [4,36].

Hormone-producing Leydig cells also express VDR and VDME, indicating a direct link between VD and the synthesis of sex steroids [4]. Indeed, individuals with testicular problems were found to have lower systemic 25(OH)D levels than healthy controls due to reduced local CYP2R1 expression in Leydig cells [37]. 

The male reproductive function is mediated by the hypothalamic–pituitary–gonad axis. Both the VDR and the enzymes that metabolize VD are found in the central organs of this system. In the brain, VDR and 1α-hydroxylase are most strongly expressed in the substantia nigra and hypothalamus. VDR expression at the gene and protein level has also been confirmed in the pituitary gland [38,39].

Animal studies further support the role of VD in reproduction, revealing a link between VD deficiency and reduced fertility or gonadal dysfunction. VDR has been discovered in the smooth muscles of the epididymis, spermatogonia, and Sertoli cells of male rodents, indicating a role for vitamin D in spermatogenesis and sperm maturation in rats [40]. Early studies have shown that male rats fed a vitamin D-deficient diet have lower fertility than animals fed a vitamin D-rich diet. The number of successful mating, expressed as the presence of sperm in the female reproductive tract, decreased by 45–55% in male rats deficient in VD [41,42]. Additionally, the fertility of normal female rats inseminated by vitamin D-deficient males was reduced by 73% [42]. In both studies, male rats fed a diet deficient in VD showed changes in testicular histology. Males with hypovitaminosis had lower testicular and epididymal sperm counts, Sertoli cell dysfunction, fewer Leydig cells, and degenerative changes in the reproductive epithelium [41,42]. Similar results were obtained in VDR knockout mouse models. Male mice with the VDR null mutation had lower sperm count and motility, as well as testicular histological abnormalities, including a dilated lumen of the seminiferous tubules, a thinner epithelial cell layer, and decreased spermatogenesis [43]. These findings supported the effect of VD on testicular function and, by extension, male infertility.

## 3. Materials and Methods

A review of relevant literature was conducted using the MEDLINE, Cochrane, and Web of Science databases. The following keywords were used in the search: vitamin D, cholecarciferol, ergocarciferol, vitamin D levels, male fertility, male infertility, semen, sperm, sex hormones, testosterone, estradiol, follicle stimulating hormone, luteinizing hormone, sex hormone binding globulin, inhibin b. During the search, individual keywords and their combinations were typed using AND, OR, or both. The literature search covered papers published from January 2011 to June 2022. Only English-language scientific papers were taken into account. All published randomized clinical trials, retrospective, prospective, observational, and comparative human studies were included, while case reports, comments/letters to the editor, and reviews were omitted. The analysis did not include any in vitro or animal investigations. Title and abstracts were assessed, and articles were classified according to predefined inclusion and exclusion criteria. The eligibility of each article found was assessed independently by two reviewers, and excluded records were verified by another. Full text of selected articles was obtained for further review. Two reviewers independently assessed the quality of the full-text papers describing the strength and level of confidence of the results using the levels of evidence published by The Oxford Centre for Evidence-Based Medicine (CEBM) in 2009 [44]. An Excel file was created containing the following data from selected publications: name of the first author of each study, year of publication, country, sample size, age of participants, health status, study design, variables used in adjustments, reported semen, and sex hormones rates. One reviewer extracted data, the correctness of which was checked by other reviewers, and all discrepancies were resolved through discussion. This review was registered in the INPLASY platform of registered systematic review and meta-analysis protocols (INPLASY2022110151) and followed the Preferred Reporting Items for Systematic Reviews and Meta-Analyses (PRISMA statement) guidelines [45].

## 4. Results

**Figure 2:**
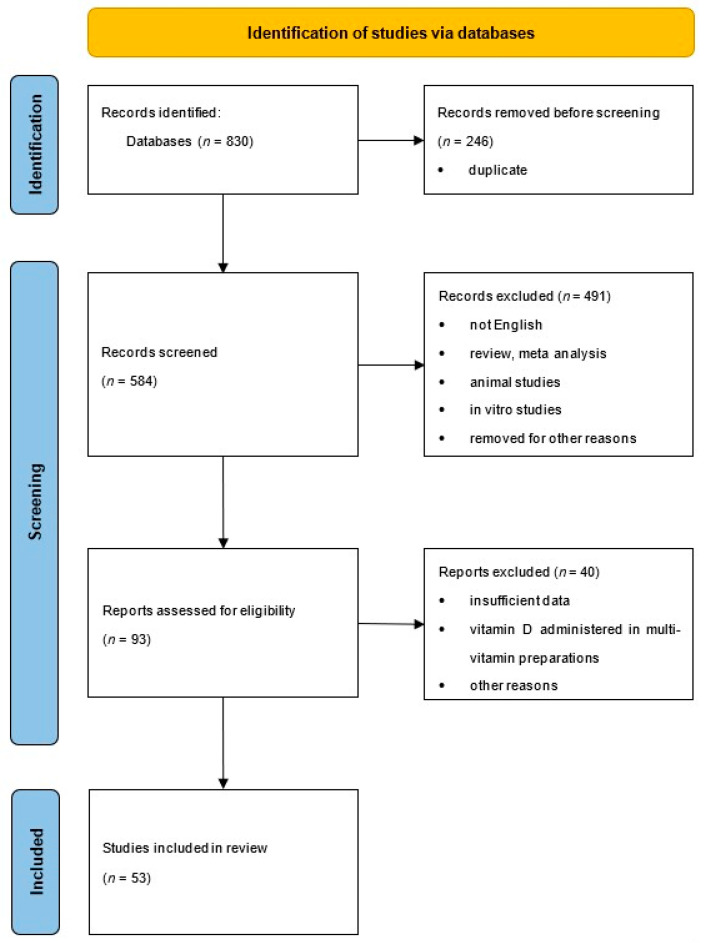
PRISMA flow chart of the literature search and screening process.

An initial database search found 830 studies. After applying the eligibility criteria to the titles and abstracts, 193 articles were eligible for the full-text review. This number was reduced to 53 eligible studies by a literature search and screening process. The selection approach is shown in the PRISMA chart (Figure 2). All detailed data collected from the studies are presented in Table 1, Table 2, Table 3 and Table 4. Studies on VD included patients from 24 different nations: Argentina, Austria, Bangladesh, Brazil, China, Denmark, Egypt, Germany, Holland, India, Iran, Ireland, Italy, Jordan, Malaysia, Norway, Pakistan, Poland, Qatar, Slovakia, South Korea, Spain, Turkey, and the USA. The age of the participants ranged from 18 to 97 years. Five studies focused on young people, six on elderly males, and the remaining studies examined middle-aged men or patients of various ages. The analyzed participants were drawn from the general population (*n* = 16), were patients of andrology clinics/had semen abnormalities (*n* = 27), or were selected on the basis of other criteria (*n* = 10). 

Out of the included 53 articles, 32 focused on the effect of VD on sperm parameters (Table 1 and Table 2) and 34 discussed the effect on sex hormones (Table 3 and Table 4). Furthermore, 36 studies were classified as observational and 17 as interventional. Various techniques were used to assess the level of VD in the serum: chemiluminescence immunoassay (*n* = 13), electrochemiluminescence (*n* = 7), enzyme-linked immunosorbent assay (*n* = 10), isotope dilution liquid chromatography tandem mass spectrometry (*n* = 8), radioimmunoassay (*n* = 5), high performance liquid chromatography (*n* = 4), competitive protein-binding assay (*n* = 2), enzyme-linked fluorescent assay (*n* = 1), liquid chromatography mass spectrometry (*n* = 1), chemiluminescent microparticle immunoassay (*n* = 1). Four studies did not report the method of measurement.

### 4.1. Vitamin D and Semen Parameters: Observational Studies

Of the included articles, 23 investigated the relationship between VD levels and semen quality using observational studies. All are cross-sectional studies. The studies are summarized in Table 1.

VD levels can be seen to have a beneficial effect on sperm quality, as indicated by a positive correlation between VD serum levels and total and/or progressive sperm motility [35,46,47,48,49,50,51,52,53,54,55,56,57,58,59,60]. This correlation was significant in men from the general population [35,46], but also in patients of andrology clinics/men with semen abnormalities [47,48,49,50,51,52,53,54,55,56,57,58,59,60]. However, seven studies failed to link VD to sperm motility or returned statistically insignificant results [61,62,63,64,65,66,67]. 

In contrast, the existence of a correlation between VD levels and sperm count and sperm morphology is uncertain. Only eight studies showed a positive relationship between sperm concentration/total number and VD levels [46,52,53,54,55,57,58,60], and ten publications confirmed a correlation between VD levels and normal sperm morphology [35,47,51,52,53,54,56,57,58]. Only one study found that low VD levels are not a risk factor for poor semen quality in a population of young healthy men, although high VD levels were unexpectedly associated with a lower percentage of normal sperm morphology. However, after the adjustment for potential confounders, the results were found to be insignificant [61].

In contrast, a cross-sectional study of 170 men revealed an association between U-shaped VD levels and semen parameters, showing that both low and high VD levels are associated with poor semen quality [46]. In this study, men with 25(OH)D ≥ 50 ng/mL (≥125 nmol/mL) had lower sperm concentration, sperm progressive motility, sperm morphology, and total progressive motile sperm count compared to men with 20 ng/mL ≤ 25(OH)D < 50 ng/mL (50 nmol/mL ≤ 25(OH)D < 125 nmol/mL) [46].

The first study on the relationship between serum VD levels and sperm DNA fragmentation in humans appeared in 2018. Azizi et al. [51] postulated that there may be a link between serum 25-OHD and reactive oxygen species (ROS) in semen and sperm DNA fragmentation. The results suggest that increasing 25(OH)D levels are associated with a decrease in apoptotic cells and ROS, however, the relationship was not significant. Since then, only two studies have been conducted that showed a significant negative correlation between VD and sperm DNA fragmentation [53,58].

### 4.2. Vitamin D and Semen Prameters: Interventional Studies

The last few years have seen several studies on the effect of VD supplementation on sperm parameters. This review collated ten such studies, six of which were randomized clinical trials, two prospective interventional studies, one longitudinal observation study, and one case control. The summary and general effect of VD supplementation on semen parameters in clinical trials are presented in Table 2.

Many studies have shown an improvement in motility, especially progressive after supplementation with VD [68,69,70,71,72,73]. However, only four of the intervention studies demonstrated any efficacy in increasing sperm concentration [69,70,71,73]. Only one study found administration of VD to improve sperm morphology [70]. The effects do not seem to be dependent on the length of VD supplementation: both short-term (2–3 months) and long-term (5–6 months) supplementation was able to improve patient outcomes [68,69,70,71,72,73,74,75]. In addition, studies of the same length differed in their results: both positive results [71,72,73] and no changes [75,76] were obtained after three-month supplementation with VD. These differences may be due to adjustment for potential confounders; however, all of the three-month studies were unadjusted for any covariates.

**Table 1:** Summary of Observational Studies between Vitamin D and Semen Quality Parameters in Men.

Study	Country	Study Design	Number of Patients	Age (Years) ^1^	Characteristics of Patients	Vitamin D Measurement Method	Sperm Parameters	Adjustment	Level of Evidence
2022 Güngör et al. [58]	Turkey	Cross-Sectional	108	F: 33.2 ± 4.1 I: 34.7 ± 4.0	Fertile and infertile men	ECLIA	SC↑ TM↑ NM↑ DF↓	Unadjusted	3b
2022 Holzer et al. [59]	Argentina	Cross-Sectional	56	20–45	Andrological patients	CLIA	SC→ TM↑ PM↑ NM→	Unadjusted	4
2022 Kamal et al. [67]	Egypt	Cross-Sectional	100	35.0 ± 8.4 (20–50)	Andrological patients	ELISA	SC→ PM→ NM→	Unadjusted	4
2022 Rezayat et al. [60]	Iran	Cross-Sectional	114	F: 34.9 ± 13.5 I: 34.6 ± 12.2 (20–59)	Fertile and infertile men	ELISA	SC↑ TM↑ NM→	Unadjusted	3b
2021 Ciccone et al. [57]	Brazil	Cross-Sectional	260	SA: 38.7 ± 8.5 NS: 38.0 ± 8.8 (18–60)	Men with semen abnormalities and normozoospermic men	ECLIA/HPLC	SC↑ TM↑ PM↑ NM↑	Unadjusted	3b
2021 Hajianfar et al. [54]	Iran	Cross-Sectional	350	34.8 ± 0.4 (20–50)	Andrological patients	ECLIA	SC↑ TM↑ NM↑	Age, Educational Status, Smoking, Alcohol Consumption, Job, Varicocele, BMI, Total Energy Intake	4
2021 Hussein et al. [55]	Egypt	Cross-Sectional	100	F: 31.4 ± 8.9 (19–58) I: 32.8 ± 6.9 (18–50)	Fertile and infertile men	ELFA	SC↑ PM↑ NM→	Unadjusted	3b
2021 Kumari et al. [56]	India	Cross-Sectional	224	18–45	Men with semen abnormalities and normozoospermic men	CMIA	SC↑ TM↑ PM↑ NM↑	Unadjusted	3b
2020 Derakhshan et al. [53]	Iran	Cross-Sectional	70	SVD: 33.7 ± 5.7 IVD: 36.3 ± 6.5 (18–60)	Andrological patients	HPLC	SC↑ TM↑ PM↑ NM↑ DF↓	Unadjusted	4
2020 Rudnicka et al. [66]	Spain	Cross-Sectional	198	18–23	Young men from general population	CLIA	SC→ TM→ NM→	Age, BMI, Smoking, Physical activity, Season, Ejaculation Abstinence Time, Time to Start of Semen Analysis	2b
2019 Jueraitetibaike et al. [65]	China	Cross-Sectional	222	30	Andrological patients	ECLIA	SC→ TM→ PM→ NM→	Unadjusted	4
2018 Azizi et al. [51]	Iran	Cross-Sectional	62	NS: 34.1 ± 1.2 OAT: 33.0 ± 0.7	Men with semen abnormalities and normozoospermic men	CLIA	SC→ TM↑ NM↑ DF→	Unadjusted	3b
2018 Jóźków et al. [64]	Poland	Cross-Sectional	177	24.6 ± 3.6 (20–35)	Young men from general population	ECLIA	SC→ PM→	Smoking, Alcohol Consumption, Carrying a Telephone in a Pants Pockets, BMI, WHR, Caffeine Consumption, Physical Activity	2b
2018 Rehman et al. [52]	Pakistan	Cross-Sectional	313	25–55	Fertile and infertile men	Na	SC↑ TM↑ NM↑	Vitamin D adjusted with BMI	3b
2017 Abbasihormozi et al. [49]	Iran	Cross-Sectional	278	33.5 ± 4.8 (20–50)	Men with oligoasthenoteratozoospermia and normozoospermic men	ECLIA	SC→ TM↑ PM→ NM→	Age, BMI, Season	3b
2017 Tirabassi et al. [50]	Italy	Cross-Sectional	104	33.1 ± 4.8	Andrological patients	CLIA	SC→ TM↑ PM↑ NM→	Age, BMI, PTH, Varicocele	4
2016 Blomberg Jensen et al. [48]	Denmark	Cross-Sectional	1189	34.3	Andrological patients	ID-LC-MS/MS	SC→ TM↑ PM↑ NM→	Age, BMI, Smoking, Season, Abstinence, Time from Collection, Free Testosterone, Estradiol, Total Estradiol, Testosterone/Estradiol Ratio	2b
2016 Neville et al. [62]	Ireland	Cross-Sectional	73	37.4 ± 4.4	Men from couples undergoing IVF/ICSI	CPBA	SC→ TM→ PM→ NM→	Unadjusted	4
2016 Zhu et al. [63]	China	Case-Control	265	F: 28.22 ± 0.5 O: 28.8 ± 0.8 A: 28.3 ± 0.6 OA: 27.7 ± 0.6 AZ: 27.2 ± 0.6 NS: 28.4 ± 0.4	Fertile and infertile men	ELISA	SC→ TM→ PM→	Unadjusted	3b
2012 Hammound et al. [46]	USA	Cross-Sectional	147	29.0 ± 8.5 (18–67)	General population	CLIA	SC↑ PM↑ NM→	Age, BMI, Season, Alcohol Consumption, Smoking	2b
2012 Yang et al. [47]	China	Cross-Sectional	559	20–40	Fertile and infertile men	ELISA	TM↑ NM↑	Season, Abstinence, Time from Collection	3b
2011 Blomber Jensen et al. [35]	Denmark	Cross-Sectional	300	19.0	General population	ID-LC-MS/MS	SC→ TM↑ PM↑ NM↑	Abstinence, Season, Medication, Fever, Time from Ejaculation to Motility Assessment	2b
2011 Ramlau-Hansen et al. [61]	Denmark	Cross-Sectional	307	18–21	Young men from general population	ID-LC-MS/MS	SC→ TM→ NM→	Season, History of Diseases of the Reproductive Organs, Smoking, Maternal Smoking During Pregnancy, Maternal Alcohol During Pregnancy, Abstinence, Spillage During Collection of The Sample	2b

Abbreviations: A: asthenospermia; AZ: azoospermia; CLIA: chemiluminescence immunoassay; CMIA: chemiluminescent microparticle immunoassay; CPBA: competitive protein-binding assay; DF: DNA fragmentation; ECLIA: electrochemiluminescence; ELFA: enzyme-linked fluorescent assay; ELISA: enzyme-linked immunosorbent assay; F: fertile; HPLC: high performance liquid chromatography; I: infertile; ID-LC-MS/MS: isotope dilution liquid chromatography tandem mass spectrometry; IVD: insufficient vitamin D; Na: Not available; NM: normal morphology; NS: normozoospermic; O: oligospermia; OA: oligoasthenospermia; OAT: oligoasthenoteratozoospermic; PM: progressive motility; RIA: radioimmunoassay; SA: seminal abnormalities; SC: sperm concentration; SVD: sufficient vitamin D; TM: total motility. → no correlation; ↑ positive correlation; ↓ negative correlation. ^1^ Age values are presented as mean ± standard deviation, mean ± standard deviation (range), range only, or median only.

**Table 2:** Summary of Interventional Studies between Vitamin D and Semen Quality Parameters in Men.

Study	Country	Study Design (Duration)	Vitamin D Dose	Number of Patients	Age (Years) ^1^	Characteristics of Patients	Vitamin D Measurement Method	Sperm Parameters	Adjustment	Level of Evidence
2022 Padmapriya et al. [73]	India	DBRCT (3 months)	28,000 IU of VD3 weekly	120	30–40	Men with oligoasthenoteratozoospermia	Na	SC↑ TM↑ PM↑	Unadjusted	1b
2021 Bartl et al. [70]	Slovakia	Prospective interventional study (6 months)	17,500 IU of VD3 weekly	34	36.6	Infertile men	HPLC	SC↑ PM↑ NM↑	Unadjusted	2b
2021 Begum et al. [71]	Bangladesh	Prospective interventional study (3 months)	40,000 IU of VD3 weekly for six weeks, 14,000 IU of VD3 weekly for another six weeks	110	33.2 ± 5.8 (25–45)	Men with asthenozoospermia and vitamin D deficiency	CLIA	SC↑ TM↑ PM↑	Unadjusted	2b
2021 Gheflati et al. [76]	Iran	DBRCT (3 months)	50,000 IU of VD3 weekly for eight weeks and one maintenance dose of 50,000 IU for another four weeks	44	18–45	Men with asthenozoospermia	ELISA	SC→ PM→ NM→	Unadjusted	1b
2021 Maghsoumi-Norouzabad et al. [72]	Iran	TBRCT (3 months)	28,000 IU of VD3 weekly	86	VD: 35.1 ± 5.5 P: 34.4 ± 5.1	Men with asthenozoospermia	ELISA	SC→ TM↑ PM↑ NM→	Unadjusted	1b
2020 Amini et al. [75]	Iran	TBRCT (3 months)	50,000 IU of VD3 weekly for eight weeks and one maintenance dose of 50,000 IU for another four weeks	62	35–39	Men with semen abnormalities	ELISA	SC→ TM→ PM→ NM→	Unadjusted	1b
2020 Wadhwa et al. [69]	India	Prospective interventional study (6 months)	60,000 IU of VD3 and 3500 mg of calcium weekly	60	30.6 ± 4.0 (23–40)	Men with asthenozoospermia, oligozoospermia, or both	Na	SC↑ PM↑	Unadjusted	2b
2018 Blomberg Jensen et al. [74]	Denmark	TBRCT (5 months)	single dose of 300,000 IU of VD3, then9800 IU of VD3 and 3500 mg of calcium weekly	330	34.8 ± 6.6	Men with semen abnormalities	ID-LC-MS/MS	SC→ TM→ PM→ NM→	Unadjusted	1b
2017 Alzoubi et al. [68]	Jordan	Case-Control (2 months)	35,000 IU of VD3 weekly	34	20–45	Men with semen abnormalities	ELISA	SC→ TM↑ PM↑ NM→	Unadjusted	2b

Abbreviations: CLIA: chemiluminescence immunoassay; DF: DNA fragmentation; ELISA: enzyme-linked immunosorbent assay; HPLC: high performance liquid chromatography; ID-LC-MS/MS: isotope dilution liquid chromatography tandem mass spectrometry; IVD: insufficient vitamin D; Na: Not available; NM: normal morphology; P: placebo; PM: progressive motility; SC: sperm concentration; SVD: sufficient vitamin D; TM: total motility; VD: vitamin D; → no change; ↑ increase. ^1^ Age values are presented as mean ± standard deviation, mean ± standard deviation (range), mean only, range only, or median only.

### 4.3. Vitamin D and Sex Hormones: Observational Studies

Both VDR and the enzymes metabolizing VD are expressed in Leydig cells [4], suggesting that VD plays a direct role in regulating steroidogenesis. Twenty-two of the human studies of the association between 25(OH)D and sex hormones are summarized in Table 3. 

The effect of VD on serum levels of male sex hormones has been analyzed in several studies, but with inconsistent results. Most observational studies indicate that serum 25(OH)D3 levels were not associated with circulating total testosterone (TT) or free testosterone (FT) levels [46,47,48,49,50,59,61,77,78,79,80,81], with exceptions in seven studies [52,57,60,82,83,84,85]. In addition, some of these studies revealed a significant association between 25(OH)D and sex hormone binding globulin (SHBG) [48,59,61,77,79]. In addition, two studies found a negative association with FT and a positive relationship with SHBG [48,79]. In such situations, VD deficiency may indirectly affect the hormonal state by modulating the bioavailable fraction of testosterone.

The relationship between VD and testosterone may be age dependent. The majority of studies on older men (over 50) show a positive relationship between 25(OH)D levels and testosterone [82,83,84,86,87]. However, this association disappeared after adjusting for confounding factors such as comorbidities [86]. No association was found between 25(OH)D and testosterone in men aged under 35 [46,48,61,88]. This suggests that the relationship between VD and testosterone may be influenced by age-varying factors such as an increase of SHBG level, as well as age-related comorbidities including endocrine pathologies. 

Moreover, studies by Zhao et al. [79] and Blomberg Jensen et al. [48] revealed a negative correlation between 25(OH)D3 and estradiol (E2) level. In contrast, two other studies showed a positive relationship with E2 [83,87]. However, less promising results were noted for other hormones; none of the eligible studies discovered any connection between VD and FSH, and one study found a negative association between LH and VD [52]. In addition, only one study investigated the correlation between VD and inhibin B, and it was found to be positive [48].

### 4.4. Vitamin D and Sex Hormones: Interventional Studies

The search also encompassed the potential impact of VD supplementation on male hormones. The results are given in Table 4. 

Unfortunately, intervention studies have not achieved any unequivocal findings on the effects of VD on sex hormones. The effects seemed to be variable and dependent on the length of VD supplementation. Short-term (1.5–4 months) supplementations were not capable to influence circulating levels of TT [72,75,76,89,90,91,92]. Long-term (6–12 months) VD supplementation in different age groups may generate a significant increase in TT [93] and/or FT and SHBG [94] or no change [70,95]. However, even longer (36 months) interventions did not show a positive effect with a decrease in FT levels being noted [96]. One study found VD therapy to have a positive effect on estradiol levels [90] while another found a negative one [93]. SHBG has been found to decrease with VD supplementation [75,90]. The other hormones were not affected by supplementation or were not taken into account in the studies. Ergocalciferol (VD2) supplementation was only used in one study [93]. However, the findings do not indicate that VD3 supplementation yielded any significant benefits in this regard.

**Table 3:** Summary of observational studies between vitamin D and sex steroids in men.

Study	Country	Study Design	Number of Patients	Age (Years) ^1^	Characteristics of Patients	Vitamin D Measurement Method	Hormones	Adjustment	Level of Evidence
2022 Holzer et al. [59]	Argentina	Cross-Sectional	56	20–45	Andrological patients	CLIA	TT→ FT→ E2→ LH→ FSH→ SHBG ↑	Unadjusted	4
2022 Rezayat et al. [60]	Iran	Cross-Sectional	114	F:34.9 ± 13.5 I:34.6 ± 12.2 (20–59)	Fertile and infertile men	ELISA	TT↑ LH→ FSH→	Unadjusted	3b
2022 Talebi et al. [81]	Iran	Cross-Sectional	220	34.5 ± 5.6 (20–45)	Men with semen abnormalities	Na	TT→ FT→ LH→ FSH→	Unadjusted	4
2021 Ciccone et al. [57]	Brazil	Cross-Sectional	260	SA: 38.7 ± 8.5NS: 38.0 ± 8.8 (18–60)	Men with semen abnormalities and normospermic men	ECLIA/HPLC	TT↑	Unadjusted	3b
2021 Książek et al. [80]	Poland	Cross-Sectional	176	18–35	Active young men from general population	ECLIA	TT→ FT→ LH→ FSH→ SHBG →	Age, BMI, Smoking, Alcohol Consumption, WHR	2b
2019 Chen et al. [97]	China	Cross-Sectional	4254	18–93	General population	CLIA	TT↑	Age, Economic Status, Smoking, BMI, Hypertension, Diabetes	2b
2018 Rehman et al. [52]	Pakistan	Cross-Sectional	313	25–55	Fertile and infertile men	Na	TT↑ LH↓ FSH→	Vitamin D adjusted with BMI	3b
2017 Abbasihormozi et al. [49]	Iran	Cross-Sectional	278	33.5 ± 4.8 (20–50)	Men with oligoasthenoteratozoospermia and normospermic men	ECLIA	TT→ FT→ LH→ FSH→	Age, BMI, Season	3b
2017 Tirabassi et al. [50]	Italy	Cross-Sectional	104	33.1 ± 4.8	Andrological patients	CLIA	TT→	Age, BMI, PTH, Varicocele	4
2017 Zhao et al. [79]	USA	Cross-Sectional	3017	62.1 ± 10.2 (45–84)	General population	HPLC–tandem mass spectrometry	TT→ FT↓ E2↓ SHBG ↑	Age, Race/Ethnicity, Study Site, BMI, Smoking, Education, Self-Reported Good Health Status, Intentional Physical Activity, Diabetes, Systolic Blood Pressure, Use of Antihypertensive Medications, eGFR, Total Cholesterol, HDL Cholesterol, Use of Lipid Lowering Medication Usage, C-Reactive Protein	2b
2016 Anic et al. [85]	USA	Cross-Sectional	1633	≥20	General population	RIA	TT↑ FT→ SHBG ↑	Age, Race/Ethnicity, % Body Fat, Smoking	2b
2016 Barbonetti et al. [78]	Italy	Cross-Sectional	49	47.5 ± 17.3	Men with chronic spinal cord injury	CLIA	TT→ FT↑	Age, Smoking, Alcohol Consumption, Coexisting Illness, Homeostatic Model Assessment of Insulin Resistance, Functional Independence Degree (Barthel Index), BMI, Weekly Leisure Time Physical Activity	4
2016 Blomberg Jensen et al. [48]	Denmark	Cross-Sectional	1189	34.3	Andrological patients	ID-LC-MS/MS	TT→ FT↓ E2↓ LH→ FSH→ SHBG ↑ INHB↑	Age, BMI, Smoking, Season, Abstinence, Time from Collection, Free Testosterone, Estradiol, Total Estradiol, Testosterone/Estradiol Ratio	2b
2016 Rafiq et al. [84]	Holland	Cohort Study	643	65–89	Older men from general population	CPBA	TT↑ FT→ E2→ LH→ FSH→ SHBG →	Age, BMI, Season, Alcohol Consumption, Smoking, Number of Chronic Diseases, Physical Function, Serum Creatinine	2b
2015 Chin et al. [77]	Malaysia	Cross-Sectional	382	43.5 ± 15.5	General population	ELISA	TT→ FT→ SHBG ↑	Age, BMI, Race/Ethnicity	2b
2015 Tak et al. [82]	South Korea	Cross-Sectional	652	56.7 ± 7.9 (40–80)	Men over 40 years old from general population	CLIA	TT↑ FT↑	TT: Age, BMI, Waist Circumference, % Body Fat, Fasting Plasma Glucose, Diabetes, Dyslipidemia. FT: Age, Total Muscle Mass, Smooth Muscle Mass, Total Cholesterol, Diabetes, Dyslipidemia, Alcohol Consumption	2b
2015 Wang et al. [83]	China	Cross-Sectional	2854	53.0 ± 13.5	General population	CLIA	TT↑ FT→ E2↑ LH→ FSH→ SHBG →	Age, BMI, Residence Area, Economic Status, Smoking, HOMA-IR, Diabetes, Systolic Pressure	2b
2014 Lerchbaum et al. [88]	Austria	Cross-Sectional	225	35	Middle-aged men from general population	ID-LC-MS/MS	TT→ FT→ E2→ LH→ FSH→ SHBG →	Unadjusted	4
2012 Hammound et al. [46]	USA	Cross-Sectional	147	29.0 ± 8.5 (18–67)	General population	CLIA	TT→ FT→ E2→ LH→ FSH→ SHBG →	Age, BMI, Season, Alcohol Consumption, Smoking	2b
2012 Lee et al. [86]	Europe	Cross-Sectional	3369	40–79	General population	RIA	TT→ FT→ E2→ LH→ FSH→ SHBG →	Age, Centre, BMI, Smoking, Alcohol Consumption, Physical Activity, Physical Function, Heart Conditions, Hypertension, Diabetes, Depression	2b
2012 Nimptsch et al. [87]	USA	Cross-Sectional	1362	40–75	Participants selected for a nested case–control study on prostate cancer	RIA	TT↑ FT↑ E2↑	Age, Batch, BMI, Season, Geographical Region, Smoking, Physical Activity, Time of Blood Collection. TT Further Adjusted for SHBG	3b
2011 Ramlau-Hansen et al. [61]	Denmark	Cross-Sectional	307	18–21	Young men from general population	ID-LC-MS/MS	TT→ E2→ LH→ FSH→ SHBG ↑	Season, History of Diseases of the Reproductive Organs, Smoking, Maternal Smoking During Pregnancy, Maternal Alcohol During Pregnancy, Abstinence, Spillage During Collection of the Sample	2b

Abbreviations: BMI: body mass index; CLIA: chemiluminescence immunoassay; CPBA: competitive protein-binding assay; E2: estradiol; ECLIA: electrochemiluminescence; ELISA: enzyme-linked immunosorbent assay; F: fertile; FSH: follicle stimulating hormone; FT: free testosterone; HOMA-IR: homeostatic model assessment –insulin resistance; HPLC: high performance liquid chromatography; I: infertile; ID-LC-MS/MS: isotope dilution liquid chromatography tandem mass spectrometry; INHB: inhibin B; LH: luteinizing hormone; Na: Not available; NS: normozoospermic; PTH: parathormone; RIA: radioimmunoassay; SA: seminal abnormalities; SHBG: sex hormone binding globulin; TT: total testosterone; WHR: waist-to-hip ratio. → no correlation; ↑ positive correlation; ↓ negative correlation. ^1^ Age values are presented as mean ± standard deviation, mean ± standard deviation (range), range only, or median only. In one study, the age of patients was presented as greater than/equal to 20 (≥20).

**Table 4:** Summary of Interventional Studies between Vitamin D and Sex Steroids in Men.

Study	Country	Study Design (Duration)	Vitamin D Dose	Number of Patients	Age (Years) ^1^	Characteristics of Patients	Vitamin D Measurement Method	Hormones	Adjustment	Level of Evidence
2021 Bartl et al. [70]	Slovakia	Prospective interventional study (6 months)	17,500 IU of VD3 weekly	34	36.6	Infertile men	HPLC	TT→ FT→ E2→ LH→ FSH→ SHBG →	Unadjusted	2b
2021 Gheflati et al. [76]	Iran	DBRCT (3 months)	50,000 IU of VD3 weekly for eight weeks and one maintenance dose of 50,000 IU for another four weeks	44	18–45	Men with asthenozoospermia	ELISA	TT→ SHBG →	Unadjusted	1b
2021 Maghsoumi-Norouzabad et al. [72]	Iran	TBRCT (3 months)	28,000 IU of VD3 weekly	86	VD:35.1 ± 5.5 P:34.4 ± 5.1	Men with asthenozoospermia	ELISA	TT→ E2→ LH→ FSH→ SHBG →	Unadjusted	1b
2021 Ulrich et al. [92]	Germany	DBRCT (3 months)	5600 IU of VD3 weekly	35	20–71	General population	LC-MS/MS	TT→	Unadjusted	1b
			Dose of VD3 adapted to patients VD serum levels	18	20–71	Hemodialysis patients	CLIA	TT→	Unadjusted	1b
2020 Amini et al. [75]	Iran	TBRCT (3 months)	50,000 IU of VD3 weekly for eight weeks and one maintenance dose of 50,000 IU for another four weeks	62	35–39	Men with semen abnormalities	ELISA	TT→ FT→ LH→ FSH→ SHBG ↓	Unadjusted	1b
2019 Lerchbaum et al. [91]	Austria	DBRCT (3 months)	20,000 IU of VD3 weekly	94	47.0 ± 12.0 (18–70)	Men with serum TT levels < 10.4 nmol/L	ID-LC-MS/MS	TT→ FT→ E2→ LH→ FSH→ SBHG→	Unadjusted	1b
2019 Zittermann et al. [96]	Germany	RCT (36 months)	28,000 IU of VD3 weekly	133	18–79	Men with advanced heart failure, mean TT level of 11.2 nmol/L	CLIA	TT→ FT**↓** SHBG →	Unadjusted	1b
2017 Canguven et al. [93]	Qatar	Prospective interventional study (12 months)	150,000 IU of VD2 weekly; when serum 25(OH)D level reached 75 nmol/L, VD dose switched to 600,000 IU every eight weeks	102	53.2 ± 10.5 (35–64)	Men with serum 25(OH)D levels < 30 ng/mL	ELISA	TT→ E2**↓** LH→	Unadjusted	2b
2017 Lerchbaum et al. [90]	Austria	DBRCT (3 months)	20,000 IU of VD3 weekly	98	18–70	Men with serum 25(OH)D levels < 75 nmol/L and TT levels ≥ 10.4 nmol/L	ID-LC-MS/MS	TT→ FT→ E2↑ LH→ FSH→ SHBG**↓**	Unadjusted	1b
2015 Heijboer et al. [89]	Holland	RCT (1.5 months)	14,000 IU of VD3 weekly	92	42–86	Male patients with chronic heart failure	ID-LC-MS/MS	TT→	Unadjusted	1b
		RCT (4 months)	4200 IU of VD3 weekly	49	71–97	Male nursing home residents	RIA	TT→	Unadjusted	1b
		RCT (4 months)	8400 IU of VD3 daily	43	20–70	Vitamin D deficient male patients	ID-LC-MS/MS	TT→	Unadjusted	1b
2013 Jorde et al. [95]	Norway	RCT (12 months)	20,000–40,000 IU of VD3 and 3500 mg of calcium weekly	129	48.9 ± 10.6 (21–70)	Men with BMI 28–47 kg/m^2^	CLIA	TT→ FT→ LH→ FSH→ SHBG →	Unadjusted	1b
		RCT (6 months)	40,000 IU of VD3 weekly	53	51.2 ± 10.0 (30–75)	Men with serum 25(OH)D levels < 50 nmol/L	LC-MS/MS	TT→ FT→ SHBG →	Unadjusted	1b
		RCT (6 months)	40,000 IU of VD3 weekly	100	53.0 ±11.1 (30–75)	Men with serum 25(OH)D levels < 55 nmol/L	LC-MS/MS	TT→ FT→ SHBG→	Unadjusted	1b
2011 Pilz et al. [94]	Austria	RCT (12 months)	23,324 IU of VD3 weekly	54	VD:49.4 ± 10.2 P:46.8 ± 12.0	Healthy, overweight men, mean TT level of 10.7 nmol/L	RIA	TT↑ FT↑	Unadjusted	1b

Abbreviations: CLIA: chemiluminescence immunoassay; E2: estradiol; ELISA: enzyme-linked immunosorbent assay; FSH: follicle stimulating hormone; FT: free testosterone; HPLC: high performance liquid chromatography; ID-LC-MS/MS: isotope dilution liquid chromatography tandem mass spectrometry; INHB: inhibin B; LC-MS/MS: liquid chromatography mass spectrometry; LH: luteinizing hormone; P: placebo; RIA: radioimmunoassay; SHBG: sex hormone binding globulin; TT: total testosterone; VD: vitamin D. → no change; ↑ increase; ↓ decrease. ^1^ Age values are presented as mean only, mean ± standard deviation, mean ± standard deviation (range), or range only.

## 5. Discussion

Little is known about the role VD plays in male reproduction, despite the identification of VDR, and its associated enzymes, in the reproductive system. Most knowledge in this area has been obtained from cross-sectional observational studies, with interventional studies only appearing recently. As such, the data provides only a vague answer. The main goal of this systematic review was to synthesize evidence showing a link between VD, sperm quality parameters and sex hormone production. Table 5 summarizes the major findings from the studies included in this review.

Four out of six studies comparing infertile and fertile men revealed that the serum vitamin D levels of infertile men were considerably lower than those of fertile men [52,55,58,60]. This finding is in line with previous meta-analysis on this topic and suggests that VD has beneficial effects on male reproduction [98]. The analysis of the correlation between VD and sperm quality parameters showed it to have the most significant impact on sperm motility. In most of the included articles, serum VD levels were significantly associated with total sperm motility [35,47,48,49,50,51,52,53,54,56,57,58,59,60] and progressive motility [35,46,48,50,53,55,56,57,59]. Sperm motility is one of the key elements affecting successful fertilization. Therefore, it is important to know the factors affecting it and the underlying mechanisms. Sperm motility, as an energy-consuming process, is strongly tied to mitochondrial adenosine triphosphate (ATP) synthesis. Mitochondrial function is regulated by many substances, one of which is cyclic adenosine monophosphate (cAMP). cAMP as a cellular second messenger acts by activating various downstream factors such as protein kinase A (PKA) [99]. Jueraitetibaike et al. [65] proposed a mechanism for increasing sperm motility by promoting ATP synthesis through the cAMP/PKA pathway, as well as an increase in intracellular ions, which is influenced by VD. In this in vitro study, sperm kinetic parameters increased after incubation with 1,25(OH)2D, and with them ATP concentrations, cAMP concentrations, PKA activity, and cytoplasmic calcium concentration also increased. This would support the experimental evidence that points to a direct non-genomic effect of VD on human sperm.

There is considerable disagreement among researchers regarding the relationship between VD levels and other sperm parameters. Fewer than half of the studies found a positive and statistically significant correlation between VD levels and sperm concentration [46,52,53,54,55,56,57,58,60] and/or morphology [35,47,51,52,53,54,56,57]. 

This is the first systematic review to examine the impact of VD on sperm DNA fragmentation; to date, only three articles on this subject appear to have been published, all of which are observational. Only two of them confirmed a negative correlation between the level of sperm DNA fragmentation and the level of VD [53,58]. In addition, in the MOXI study, where men from couples with a mild factor of male infertility were given multivitamin preparations containing, among others, vitamin D, it was not possible to find differences in sperm DNA fragmentation between men with and without vitamin D deficiency [100]. Hence, it has been suggested that the sperm nuclear matrix plays a critical role in regulating DNA fragmentation and degradation. VDR is closely related to the nuclear matrix, and VD is believed to play a significant role in stabilizing the chromosomal structure and thus protecting DNA from harm and breakage. Therefore, it is plausible that VD and its receptor act as protectors of the sperm genome [101,102]. However, this requires further research.

The evidence for the effects of VD on the production of male sex steroid hormones was less apparent. Sex steroids are produced by Leydig cells [103]. On Leydig cells, VDR and VD metabolizing enzymes are also expressed. VD, when bound to VDR, acts as a transcription factor in the promoter regions of the gene encoding the steroidogenesis enzyme. The articles mentioned in the review show an inconsistent relationship between VD and the production of sex hormones. No significant correlation was found between LH, FSH, and VD levels in any of the analyzed studies. However, some studies have found a significant correlation between VD and TT, FT, and SHBG levels. Despite evidence from several studies [93,94,104,105], a recent meta-analysis did not support the hypothesis that VD stimulates testosterone levels [106]. The exact molecular mechanism linking VD to testosterone production is still unknown. Some authors have suggested the hypothesis that genomic stimulation of osteocalcin expression by VD may play a key role in the regulation of testosterone production by the testes [107].

In men, estrogens are mainly derived from circulating androgens and metabolism is regulated by the enzyme aromatase. VD has been functionally related to estrogen signaling and VDR has been found to affect estrogen levels systemically and locally through tissue-specific regulation of aromatase [108]. However, to date, no human studies have shown a convincing causal link between VD status and estrogen production [109]. More research is needed to investigate how VD status affects not only estrogen production but also estrogen signaling, which may be another direct target of VDR.

The role of VD in the male reproductive system is still poorly understood. However, evidence from animal research that connects VD insufficiency with infertility or gonadal malfunction strengthens the significance of VD in reproduction. In a study by Zamani et al. [110], animals kept on a VD-deficient diet demonstrated profoundly impaired sperm concentration, morphology, and motility, and a significant reduction in serum testosterone levels and comparable gonadotropin levels compared to controls. The depletion of VD stores and the induction of moderate degrees of VD deficiency by dietary measures significantly impaired spermatogenesis and the microscopic testicular architecture in rats. These findings can be attributed, at least in part, to decreased androgen production. 

The first in vivo study brought attention to the strong relation between D hypovitaminosis in rats and decreased levels of circulating testosterone [111]. However, it is difficult to establish the exact mechanism of the hormonal regulation of testosterone production. Some authors support the theory that in animals, testosterone secretion may be regulated by changes in intracellular calcium homeostasis in Leydig cells. It has been proposed that testosterone synthesis as a result of VD action may be mediated by osteocalcin, a hormone generated by osteoblasts that is involved in bone metabolism [112]. Recent research has revealed the impact of the fat–VD relationship on sperm motility and mitochondrial membrane potential, with animals fed a high-fat diet demonstrating lower reproductive potential than animals fed a control diet; clearly, a high-fat diet and VD deficiency both influence sperm quality, which may therefore affect fertility [113]. Additionally, significantly increased DNA fragmentation was observed in the sperm of animals fed a diet deficient in VD and those that were obese due to diet. Based on this, it can be concluded that the observed DNA damage may be the cause of the low reproductive potential in obese men deficient in VD [104]. 

Testosterone biosynthesis is closely related to the lipid metabolism in the gonads around the testes. VD regulates the synthesis and metabolism of lipids in adipose tissue through VDR [114]. *Vdr* knockout animals had impaired testicular and perinuclear adipose tissue histology, indicating a strong relationship between lipid metabolism and VDR-mediated testosterone production. These findings support the belief that VDR plays a crucial role in controlling testicular lipid metabolism, which in turn affects testosterone production and reproduction potential in male mice [115]. Although the results of animal studies are very promising, they have not been reproduced in humans yet. It is possible that the relationship between VD and semen quality parameters noted in rodent studies is not relevant to humans, or that these associations may only be reflected in severe VD deficiency. 

The most important study examining whether VD has a direct effect on male reproductive function are randomized clinical trials with VD supplementation. Despite promising findings from animal studies and human observational studies, the results of interventional studies do not yield unequivocal conclusions. Of the 10 intervention studies reviewed, 6 showed that VD supplementation improved progressive sperm motility [68,69,70,71,72,73]. The remaining findings indicated that treatment with VD had no significant effect on changes in other sperm parameters. 

To ascertain whether VD supplementation can affect blood testosterone levels, several studies have been performed. One investigation found that treating VD-deficient individuals with VD increased testosterone levels. However, in the majority of randomized clinical trials, no substantial effect of VD supplementation on male testosterone levels was reported. These discrepancies should be regarded with caution. For example, conditions with severe VD3 deficiency are likely to respond more positively to VD3 supplementation on sperm parameters than those with mild or no deficiency. Previous studies on men found a relationship between severe VD3 deficiency and decreased fertility [48,74,116]. Moreover, there is mounting evidence that traditional VD supplementation fails to achieve appropriate 25(OH)D levels. Since VD is fat-soluble, it accumulates mostly in adipose tissue [117]. Obese men are more vulnerable to deficiency in this manner. When VD buildup in adipose tissue limits release into the systemic circulation, individuals with autoimmune disorders may require very large doses of VD treatment to boost blood 25(OH)D levels slightly [118]. Given that obese people with autoimmune disorders frequently have 25(OH)D insufficiency and decreased testosterone levels, situations like these should be examined in future studies [119,120].

The results of the studies selected for this review should be treated with caution due to some limitations. Most studies used a small sample size with high levels of heterogeneity in the study design. In addition, possible confounding factors (other causes of infertility, comorbidities, lifestyle, eating habits, age, season, ethnicity) may hinder the correct interpretation of the research. The inability to make appropriate comparisons between studies is also due to the variability in the choice of cut-off values for determining vitamin D deficiency by the authors. Another limitation that certainly influences the results are the VDBP levels and VDR polymorphisms, neither of which were addressed in the studies. Therefore, no statistical analysis of these results was performed as high heterogeneity would lead to ambiguous or unreliable results. Moreover, most of the studies included in the review are cross-sectional studies, and their nature makes it impossible to delineate a causal relationship between the variables. For these reasons, the clinical relevance of all observations made should be confirmed by further research.

## 6. Conclusions

The data presented in this review demonstrate that VD exerts a beneficial effect on sperm quality, particularly sperm motility, which is likely driven by the modulation of factors related to sperm function, namely the direct non-genomic effect of VD on sperm in men. In addition, none of the included studies have been able to exhibit a clear connection between testicular hormone production and VD levels. This evidence is mainly based on small association studies in humans and rodent models. However, there is still a lack of solid evidence from interventional studies, as these tend to be based on small sample sizes and demonstrate considerable variability regarding the optimal dose and duration of VD supplementation. Due to the increasing number of men suffering from reproductive problems and endocrine disorders, more high-quality RCTs are needed to elucidate the effects of VD supplementation on sperm parameters, especially sperm DNA fragmentation, and sex hormones.

## Figures and Tables

**Table 5:** Summary table of major conclusions.

Hypothesis		Supported	Not Supported
VD has a positive effect on sperm motility	observational studies	[35,46,47,48,49,50,51,52,53,54,55,56,57,58,59,60]	[61,62,63,64,65,66,67]
	interventional studies	[68,69,70,71,72,73]	[74,75,76]
VD increases sperm concentration	observational studies	[46,52,53,54,55,56,57,58,60]	[35,48,49,50,51,59,61,62,63,64,65,66,67]
	interventional studies	[69,70,71,73]	[68,72,74,75,76]
VD improves sperm morphology	observational studies	[35,51,52,53,54,56,57,58]	[46,48,49,50,55,59,60,61,62,65,67]
	interventional studies	[70]	[68,72,73,74,75,76]
VD reduces sperm DNA fragmentation	observational studies	[53,58]	[51]
	interventional studies	Na	Na
VD positively influences testosterone levels	observational studies	[52,57,60,78,82,83,84,85,87,97]	[46,48,59,61,77,80,81,86,88]
	interventional studies	[94]	[70,72,75,76,91,92,93,95,96]
VD affects the bioavailability of androgens by increasing the level of SHBG	observational studies	[48,59,61,77,79,85]	[46,49,50,79,80,83,84,86,88]
	interventional studies	Na	[70,72,75,76,89,90,91,95,96]

Abbreviations: Na: not applicable; SHBG: sex hormone binding globulin; VD: vitamin D.

## Data Availability

Not applicable.
